# Comment on “a novel parameter for risk stratification in patients with Takotsubo syndrome”

**DOI:** 10.1016/j.ahjo.2026.100903

**Published:** 2026-09-19

**Authors:** Güney Sarıoğlu, Yakup Yiğit

**Affiliations:** a Department of Cardiology, Ministry of Health, Battalgazi State Hospital, 44320, Malatya, Turkey; b Clinic of Cardiology, Malatya Turgut Ozal University Faculty of Medicine, 44330, Malatya, Turkey

Dear Editor,

Moady et al., who reported that a higher fibrinogen-to-albumin ratio (FAR) was associated with lower left ventricular ejection fraction (LVEF), in-hospital complications, and subsequent readmission in patients with Takotsubo syndrome (TTS) [1]. The study highlights the potential value of inflammation-related biomarkers in a condition increasingly recognized as involving systemic inflammatory responses.

An important interpretative issue, however, is whether FAR should be considered a distinct prognostic biomarker or primarily a composite expression of its two components. Fibrinogen and albumin respond in opposite directions during systemic inflammation, and albumin may also reflect nutritional status, vascular permeability, hepatic synthesis, and overall illness burden. Accordingly, a ratio can amplify separation between clinical groups without necessarily providing information beyond fibrinogen and albumin considered individually. The authors showed relationships between these components and LVEF graphically, but a direct comparison of their discrimination with FAR would clarify whether the ratio itself adds clinically meaningful information.

A second consideration concerns the distinction between a risk marker and a disease-specific marker. FAR was associated with complications even after adjustment for CRP and reduced LVEF, which is encouraging; nevertheless, this does not establish that FAR captures a TTS-specific biological pathway. Contemporary studies of inflammatory biomarkers similarly show that systemic inflammatory measures can identify cardiovascular vulnerability across diverse conditions [2]. Thus, FAR may reflect the intensity of the acute systemic response rather than a mechanism unique to TTS.

Finally, the composite in-hospital endpoint included heart failure, arrhythmia, left ventricular outflow tract obstruction, and thromboembolism—events with markedly different pathophysiology. Future studies could therefore evaluate whether FAR relates preferentially to hemodynamic decompensation or broadly to all complication types. Such phenotype-specific analyses may be more informative than a single composite association.

We believe FAR is a promising and readily available marker. The next step should be not only external validation, as the authors appropriately acknowledge, but also demonstration of incremental value over its individual components and established clinical variables, ideally using discrimination, calibration, and reclassification metrics [3], [4].

## CRediT authorship contribution statement

**Güney Sarıoğlu:** Writing – original draft, Conceptualization. **Yakup Yiğit:** Methodology, Writing – original draft.

## Author contributions

Both authors contributed to the letter's conception and the critical appraisal of the published article. G.S. conducted the initial literature review and drafted the manuscript. Y.Y. contributed to the methodological interpretation and critically revised the manuscript for important intellectual content. Both authors reviewed and approved the final version of the manuscript and agree to be accountable for the work.

## Ethical statement

This Letter to the Editor is based exclusively on the critical appraisal of a previously published article and does not involve human participants, animals, identifiable personal data, or new data collection. Therefore, ethical approval and informed consent were not required.

## Declaration of Generative AI and AI-assisted technologies in the writing process

During preparation of this manuscript, the authors used ChatGPT (GPT-5.6 Sol, OpenAI) for language refinement and to help identify relevant methodological literature. The authors independently reviewed, verified, and edited all AI-assisted output and take full responsibility for the manuscript's accuracy, integrity, and final content.

## Funding

This research received no specific grant from any funding agency in the public, commercial, or not-for-profit sectors.

## Declaration of competing interest

The authors declare no conflict of interest.

## Data Availability

No new data were generated or analyzed in this Letter to the Editor. The manuscript is based solely on the critical appraisal of a previously published article; therefore, data sharing is not applicable.
